# From biofilm tolerance to antimicrobial resistance: molecular adaptation strategies and emerging therapeutic vulnerabilities in resistant pathogens

**DOI:** 10.3389/fmicb.2026.1988464

**Published:** 2026-10-22

**Authors:** Kartik Chandra Guchhait, Subhamoy Dey, Sumita Rani Pradhan, Amiya Kumar Panda, Chandradipa Ghosh

**Affiliations:** 1 Department of Human Physiology, Vidyasagar University, Midnapore, West Bengal, India; 2 Centre for Life Sciences, Vidyasagar University, Midnapore, West Bengal, India; 3 Department of Chemistry, Vidyasagar University, Midnapore, West Bengal, India; 4 Department of Chemistry, Rani Rashmoni Green University, Hooghly, West Bengal, India; 5 Sadhu RamChand Murmu University of Jhargram, Jhargram, West Bengal, India

**Keywords:** antibiofilm therapy, antimicrobial resistance, antimicrobial tolerance, bacterial heterogeneity, biofilm, extracellular polymeric substance (EPS), quorum sensing

## Abstract

Bacterial biofilms are highly structured microbial communities which allow organisms to persist under different environmental challenges such as limitation of nutrients, oxidative stress, host immune defense, and antimicrobial agents. While antimicrobial resistance (AMR) has been well recognized as a key factor contributing to treatment failure, more and more evidence reported that biofilm-associated persistence is initially caused by antimicrobial tolerance rather than genetically encoded resistance. Tolerance is a change in physiology that enables the bacterial population to persist through a temporary exposure to an antimicrobial agent, while resistance is a stable heritable alteration that confers a decreased susceptibility to an antimicrobial agent. In biofilms, the bacteria are distributed in a heterogeneous manner in different ecological niches with different metabolic activities, oxygen gradients, stress responses and nutrient accessibility. This heterogeneity gives rise to specialized subpopulations, including dormant persisters that serve as reservoirs for further evolutionary adaptation. Biofilm development results in a selective environment that allows the pressure of antimicrobial agents to induce molecular adaptation through remodelling of extracellular polymeric substance (EPS), metabolic reprogramming, oxidative stress management, quorum sensing, cyclic di-guanosine monophosphate (c-di-GMP) signaling, efflux activation, horizontal gene transfer and diversification via mutations. Such processes form a continuum of tolerance–resistance, with short-term survival processes potentially leading to the development and fixation of resistant phenotypes. Traditional antimicrobial strategies, mainly relying on planktonic susceptibility testing, fail in the resilience of biofilms, necessitating biofilm-specific therapeutic strategies. Here, we review an integrated framework that describes the way in which bacterial biofilms move from antimicrobial tolerance to resistance evolution. We focus on strategies to intercept biofilm adaptation before stable resistance is achieved, using a combination of evolutionary microbiology, molecular regulation, single-cell biology and therapeutic innovation.

## 1 Introduction

Biofilms are one of the most successful survival tactics evolved by microorganisms. Biofilm-associated bacteria live in a structured community in which the cells are enclosed in a self-made matrix of extracellular polymeric substance (EPS), whereas free-living planktonic bacteria are not organized into a community (Sharma et al., 2026; Flemming and Wuertz, 2019). This organization protects against environmental stressors, antimicrobial compounds, immune mediated killing and nutrient fluctuation (Qureshi et al., 2026). Biofilms can therefore be considered not just a cluster of bacteria, but an ecological system of highly organized microorganisms that interact with each other through communication, competition, cooperation and evolutionary adaptation (Sharma et al., 2026). The clinical relevance of biofilms has grown significantly as they can be correlated with recalcitrant and recurring infections with medical devices, chronic wounds, respiratory infections, urinary tract infections and device associated infections (Asokan et al., 2026; Abebe, 2026). However, recent studies highlight that the bacteria in a biofilm infection are often resistant to antimicrobial treatment, as they can be in different physiological states in the biofilm, grow slowly, are protected by the presence of the matrix, and resistance genes can be exchanged among bacteria within the biofilm (Sharma et al., 2026; Biswas et al., 2025; Hall and Mah, 2017). One of the key deficits in the understanding of antimicrobial failure in the context of biofilms has been the focus on antimicrobial resistance alone. Conventional antimicrobial resistance (AMR) research has mainly concentrated on genetic mechanisms such as resistance genes, mutations, plasmids and mobile genetic elements. Yet, many of the biofilm related infections are not managed with therapeutic treatments despite a lack of conventional resistance. This observation suggests a wider biological phenomenon, antimicrobial tolerance, that can explain how organisms survive upon exposure to antimicrobials.

There is a difference between antimicrobial tolerance and antimicrobial resistance as shown in Table 1. Antimicrobial resistance is a heritable decrease in susceptibility due to genetic changes such as mutations or acquisition of resistance determinants (Ciofu et al., 2022). By comparison, antimicrobial tolerance is a transient physiological state of bacteria populations during a brief exposure to an antimicrobial agent where the bacteria survive the exposure, but without a genetic change that permanently alters the susceptibility (Brauner et al., 2016). Likewise, persistence is defined as the survival of bacteria in the presence of antimicrobials, while persister cells are phenotypic subpopulations that are able to survive lethal antimicrobial exposure and to recover when favourable conditions return (Ciofu et al., 2022; Balaban et al., 2019). Tolerance and resistance are now known to be a complex and context-dependent relationship. Tolerance does not automatically mean a requirement for an intermediate step towards resistance. However, tolerant populations can enhance the likelihood of resistance evolution because these populations allow the presence of viable bacteria during antimicrobial exposure, which can enable other evolutionary mechanisms to occur including the selection of resistant variants, genetic diversification or horizontal gene transfer (Brauner et al., 2016). Resistance can also develop by direct selection of genetic variants or by picking up resistance genes without prior tolerant state. The ecological conditions provided by biofilms might facilitate these adaptive processes through multiple, connected mechanisms such as EPS-mediated modification of antimicrobial exposure, metabolic diversification, activation of stress-response, enrichment for persisters, quorum sensing, cyclic-di-GMP regulation, efflux-mediated adaptation and horizontal gene transfer. More importantly these mechanisms are not acting alone but are linked together that will dictate the survival, adaptation or evolution of bacterial population under antimicrobial pressure. While previous researches have focussed on the mechanisms leading to biofilm resistance, antimicrobial tolerance or anti-biofilm interventions, the present review brings together these concepts in an evolutionary context. We investigate the influence of ecological organization, physiological adaptation and molecular regulation on the reversible survival states and stable resistance phenotypes. Moreover, we introduce the notion of therapeutic vulnerabilities, which are biological dependencies acquired during the adaptation to the biofilm lifestyle and which could be targeted to compromise the ability of bacteria to survive and evolve towards resistance.

**Table 1:** Conceptual distinction between antimicrobial resistance, tolerance, persistence, and adaptive resistance.

Phenotype	Definition	Genetic basis	Effect on antimicrobial susceptibility	Role in biofilm-associated survival	References
Antimicrobial resistance	A heritable reduction in antimicrobial susceptibility caused by genetic alterations	Usually involves mutations or acquisition of resistance determinants	Increased MIC and reduced antimicrobial effectiveness	Enables stable survival and propagation under antimicrobial selection	Ciofu et al. (2022), Brauner et al. (2016)
Antimicrobial tolerance	A reversible physiological state allowing survival during antimicrobial exposure without necessarily changing MIC	Usually non-heritable and phenotype-based	Survival despite antimicrobial exposure without permanent susceptibility change	Allows biofilm populations to withstand transient antimicrobial stress	Ciofu et al. (2022), Brauner et al. (2016)
Persistence	Survival of a bacterial population or subpopulation during antimicrobial treatment	Usually associated with reversible physiological adaptation	Temporary survival of a fraction of the population	Contributes to treatment failure and recurrence by maintaining viable cells	Ciofu et al. (2022), Balaban et al. (2019), Brauner et al. (2016)
Persister cells	Specialized phenotypic subpopulation capable of surviving lethal antimicrobial exposure and resuming growth after stress removal	No stable resistance mutation required	Phenotypically tolerant but genetically susceptible after recovery	Acts as a reservoir of surviving cells during antimicrobial exposure	Ciofu et al. (2022), Brauner et al. (2016)
Adaptive resistance	Temporary reduction in antimicrobial susceptibility induced by environmental signals or stress	Usually, reversible regulatory changes	Reduced susceptibility during specific conditions	Allows rapid adaptation to changing environments within biofilms	Balaban et al. (2019), Hall and Mah (2017)

## 2 Literature search strategy

The manuscript is a narrative mechanistic review designed to bring together the existing information on the evolution of antimicrobial tolerance in biofilm and the emergence of resistance. Literature was identified through searches of PubMed, Web of Science, Scopus, and Google Scholar databases. Search terms included combinations of ‘biofilm tolerance’, ‘antimicrobial resistance’, ‘persister cells’, ‘biofilm heterogeneity’, ‘horizontal gene transfer’, ‘quorum sensing’, ‘c-di-GMP signalling’, ‘efflux pumps’, and ‘anti-biofilm therapy’. Recent peer-reviewed primary research articles and high-impact reviews from the past 26 years (2000–2026) were prioritized. The studies included were chosen for their relevance to understanding the mechanisms of biofilm survival, evolutionary adaptation and therapeutic intervention. This review is not conducted in a systematic review fashion since it is not intended to provide any estimate of effect sizes or conduct a quantitative synthesis.

## 3 Biofilm adaptation under antimicrobial pressure: interactions between tolerance, persistence, and resistance evolution

Failure of antimicrobial treatment in the presence of biofilm is not simply the consequence of the presence of resistance determinants but is the outcome of the complex interplay between microbial physiology, ecological organization, environment stress and evolutionary processes (Balaban et al., 2019). Bacterial populations in biofilm environments could react to antimicrobial exposure by reversible physiological adaptation, via survival of special subpopulations or by acquiring heritable resistance mechanisms. Thus, it is important to think of adaptation to a biofilm as a dynamic process, with several potential outcomes, and not a step-by-step progression from tolerant to resistant. One of the main problems in understanding survival with antimicrobials in biofilms is that it is difficult to differentiate reversible survival phenotypes from genetically-encoded resistance. Resistance is a fixed and stable decrease in antimicrobial susceptibility and tolerance is a change that allows bacteria to survive antimicrobial exposure without necessarily changing the minimum inhibitory concentration (MIC) value (Deventer et al., 2024). These differences are significant in biofilms because many cells survive treatment without acquiring resistance genes, but in a physiological state.

### 3.1 Antimicrobial tolerance as a reversible survival phenotype

The survival of bacterial populations to a brief exposure to concentrations of antimicrobial that inhibit or kill actively growing cells is termed as antimicrobial tolerance. Tolerance is not a permanent genetic change and is usually reversible upon return to a favourable growth environment unlike resistance (Deventer et al., 2024). Biofilm environments are highly selective for tolerant states as the bacteria present within the environment are subjected to varying conditions such as limitation of nutrients, oxygen gradients, changes in pH, and varying antimicrobial exposure (Jo et al., 2022). These environmental differences produce the cells in different physiological state: actively growing cells, slow growing cells, metabolically reduced cells.

A lot of antimicrobial agents require cellular processes to be active in order to work. For instance, β-lactams must continuously synthesize the cell wall, and aminoglycosides must be transported into cells through the use of cellular energy generation and membrane potential. Thus, cells with low metabolic rate can survive the antimicrobial treatment even if they do not have classical resistance. But tolerance is not necessarily a measure of future resistance. Tolerance, on the contrary, is a survival phenotype which can affect evolutionary outcome by keeping viable bacteria during antimicrobial exposure.

### 3.2 Persister cells and population-level survival

Persister cells are a special phenotypic subpopulation that can withstand deadly exposure to antimicrobials without developing genetic resistance. Persisters are often metabolically slow, but should not be considered equal to all slow growing or dormant cells (Ciofu et al., 2022). It is important to note that there is a difference between tolerance and persistence, with tolerance generally being the ability to survive antimicrobial exposure of a population, while persistence is the survival of a subpopulation within a bacterial population (Brauner et al., 2016). Persister cells are special cells that have the ability to survive for a short period of time and grow after the antimicrobial agent is withdrawn (as illustrated in Figure 1). Biofilms facilitate the enrichment of persisters by multiple mechanisms such as metabolic adaptation, stringent response activation, toxin–antitoxin systems, stress responses and nutrient limitation. These remaining populations may be responsible for re-infections as a reservoir of bacteria may regenerate after the use of antibiotics (Santi et al., 2021). It is very important to understand that persisters are not resistant. In general, persister cells will be genetically susceptible to the antimicrobial agents when their physiological state is reversed. But their survival could mean more opportunities for evolutionary processes through continual exposure to antimicrobials.

**Figure 1:**
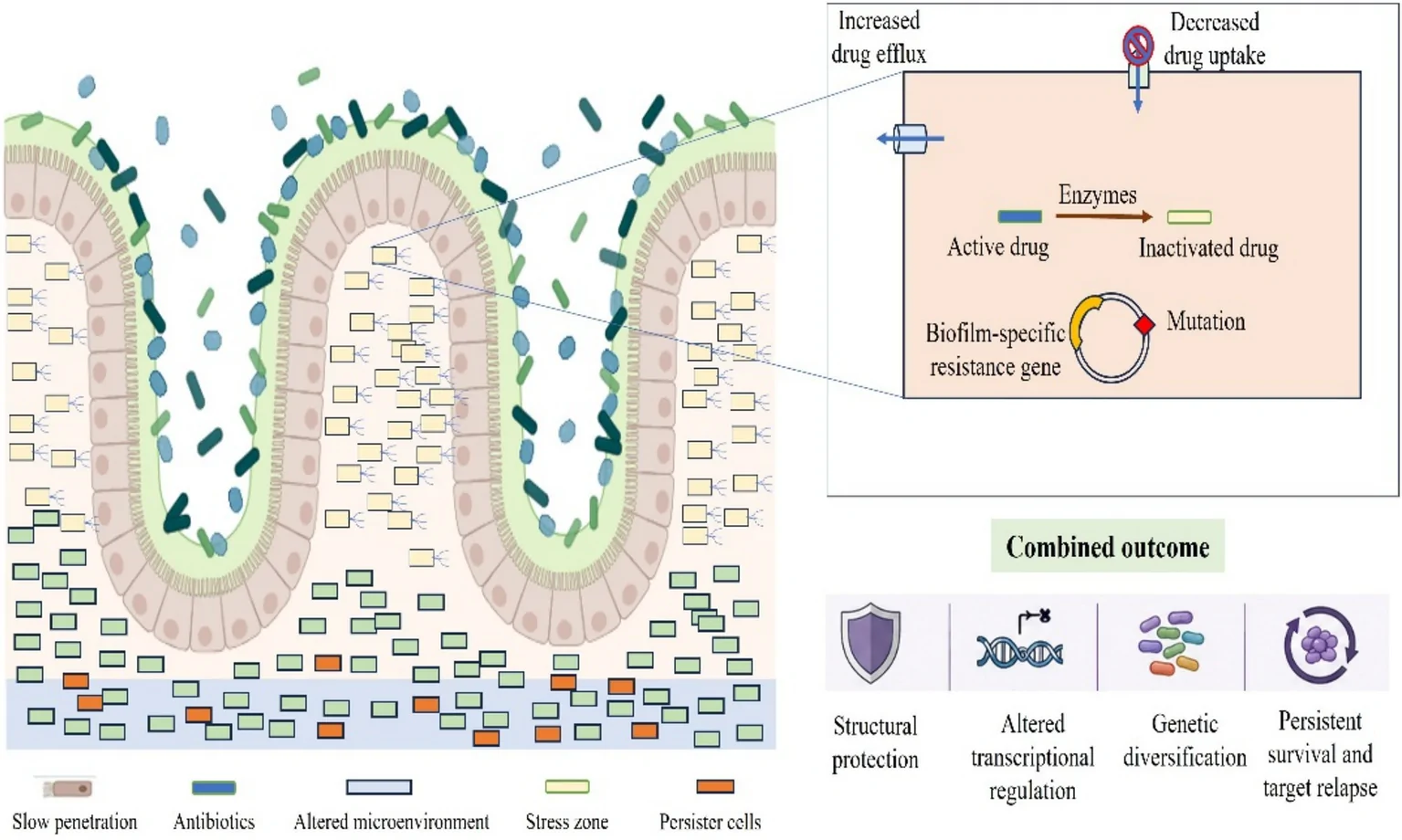
Diagrammatic representation of antibiotic resistance and persister formation in bacterial biofilm. In the presence of EPS, antibiotic penetration is slowed. In reaction to antibiotic stress, certain bacteria in the biofilm alter their behaviour. To resist biofilm eradication, the microenvironment in deeper parts is changed. Biofilms contain a higher concentration of persister cells in the altered microenvironment.

### 3.3 Conditions promoting resistance evolution in biofilms

Tolerance and persistence do not necessarily lead to antimicrobial resistance, but may affect the likelihood of resistance occurring by maintaining viable populations under selection pressure (Windels et al., 2019). Biofilm resistance evolution can take place in a number of ways:

#### 3.3.1 Selection of pre-existing resistant variants

There is genetic diversity in the population of bacteria. Resistant variants that are already present in a population may be under selection and become dominant under the antimicrobial pressure. So, resistance evolution in biofilms does not necessarily involve the development of new mutations during treatment (Trampari et al., 2021).

#### 3.3.2 Genetic diversification under stress

Biofilms may affect resistance evolution by two mechanisms: selection and diversification. It should not be assumed, however, that all the cells in the biofilm are uniformly more likely to undergo mutation due to antimicrobial exposure, oxidative stress and nutrient limitation. Rather, these conditions can lead to the enrichment of preexisting resistant variants, subpopulations that are stress adapted, or new genetic changes that arose over time (Omelaniuk et al., 2026). The role of stress-associated mutagenesis has been shown in certain experimental settings involving DNA damage responses, oxidative stress, replication fidelity and repair deficiencies, but is highly dependent on the bacterial species, environmental conditions, antimicrobial exposure, and stress intensity and duration (Ciofu et al., 2022). Thus, resistance evolution in a biofilm should be considered as a result of multiple effects of genetic variation, ecological selection, persistence of adaptive subpopulations and horizontal gene transfer, rather than as a simple effect of increased mutation rates.

#### 3.3.3 Horizontal gene transfer

Biofilms can increase the density of cells, provide close proximity of microorganisms, and hold extracellular DNA (eDNA) within the matrix, which can be important for horizontal gene transfer (Abe et al., 2020; Dale et al., 2015). The extent of the improvement in gene transfer by biofilms, however, is highly species dependent for both the bacteria and the mobile genetic element, and depends on the environmental circumstances and experimental models used. Transformation is dependent on competence mechanisms and the presence of DNA, conjugation is dependent on compatible donor-recipient interactions and mobile genetic elements, and transduction is dependent on bacteriophage biology (Abe et al., 2020; Cook and Dunny, 2014). Therefore, under certain conditions, biofilms should be regarded as ecological environments where HGT can be promoted, but not necessarily as an efficient platform for spreading resistance genes.

### 3.4 A conditional tolerance–resistance framework

The relationship between tolerance and resistance is best represented as a conditional evolutionary framework rather than a sequential pathway (as shown in Figure 2). Tolerance may create conditions that facilitate resistance evolution, but it does not represent an obligatory intermediate stage (Santi et al., 2021; Windels et al., 2019; Levin-Reisman et al., 2017).

**Figure 2:**
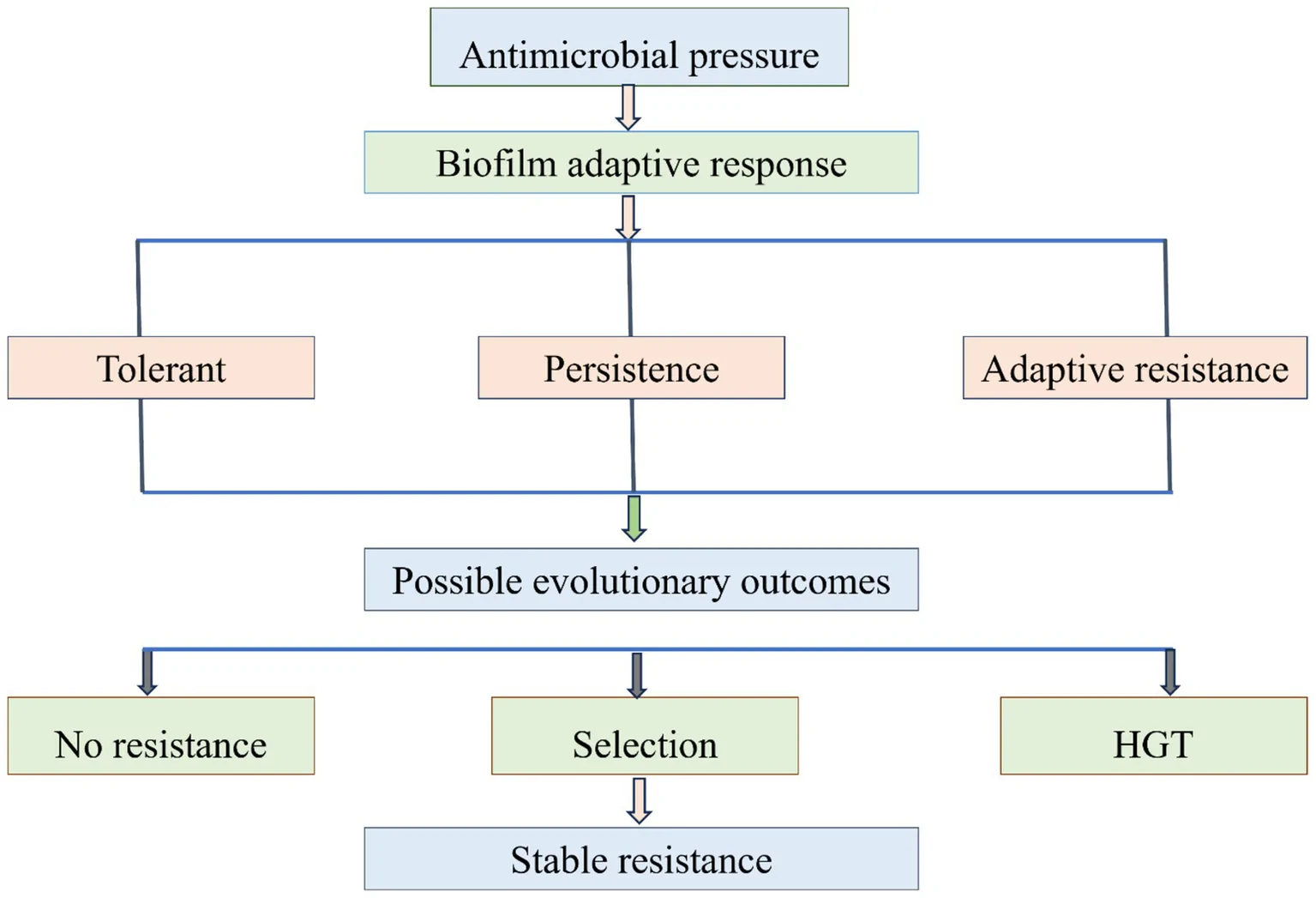
Conceptual model of biofilm survival and evolutionary outcomes under antimicrobial pressure. The model highlights multiple interacting pathways through which biofilm communities may survive antimicrobial pressure. Tolerance and persistence can increase population survival and create opportunities for evolutionary change. The transition from antimicrobial exposure to stable resistance is therefore not an obligatory or linear pathway.

## 4 Biofilm heterogeneity and molecular adaptation

At the spatial, metabolic, physiological, and regulatory levels, heterogeneity is the fundamental driver of the antimicrobial survival in a biofilm. In contrast to planktonic populations, the cells within the biofilm are in different ecological niches and therefore exposed to different levels of oxygen, nutrients, antimicrobials and metabolic byproducts. As a result, cells in the same biofilm can grow at very different rates, and have very different metabolic activities, stress responses and antimicrobial susceptibilities. This heterogeneity itself should not be interpreted as antimicrobial resistance but rather as an ecological and physiological context that might allow for tolerance, persistence, adaptive response and under proper selective conditions, evolution of resistance.

### 4.1 Spatial and metabolic heterogeneity

The three-dimensional structure of the biofilm creates oxygen, nutrient, pH and other environmental gradients. Cells near the surface of the biofilm tend to have high levels of metabolic activity because they have more access to nutrients and oxygen, while cells deeper in the biofilm may have lower levels of metabolic activity due to limitations in nutrients and oxygen. These physicochemical gradients generate spatially distinct physiological states within the biofilm, ranging from metabolically active cells in nutrient-rich regions to slow-growing or dormant populations in deeper regions (Figure 3). This spatial organization is of great importance for antimicrobial susceptibility. Many antimicrobial agents are most effective against metabolically active and dividing cells. For instance, β-lactams act by disrupting cell-wall synthesis, and aminoglycosides are taken up by energy-dependent processes. Fluoroquinolones target DNA replication and DNA topoisomerase activity. Thus, cells that are not growing or metabolizing rapidly may not be susceptible when exposed to an antimicrobial agent even though they are not genetically resistant. Metabolic heterogeneity should, therefore, be thought of more as a physiological determinant of antimicrobial response than as a resistance mechanism. Importantly, the metabolic state of each individual cell is dynamic. Environmental factors can drive shifts between growth and survival modes and enable the bacteria to adapt to varying antimicrobial and nutritional environments. The coupling of metabolic adaptation and regulatory control is also crucial. Stringent-response pathways, oxidative-stress responses, quorum sensing and c-di-GMP signalling are regulatory systems that can affect growth to survival transitions. Hence, it is not possible to consider spatial heterogeneity and molecular regulation as separate phenomena, but rather the microenvironment of the biofilm provides the signals which determine the physiological states of the cells.

**Figure 3:**
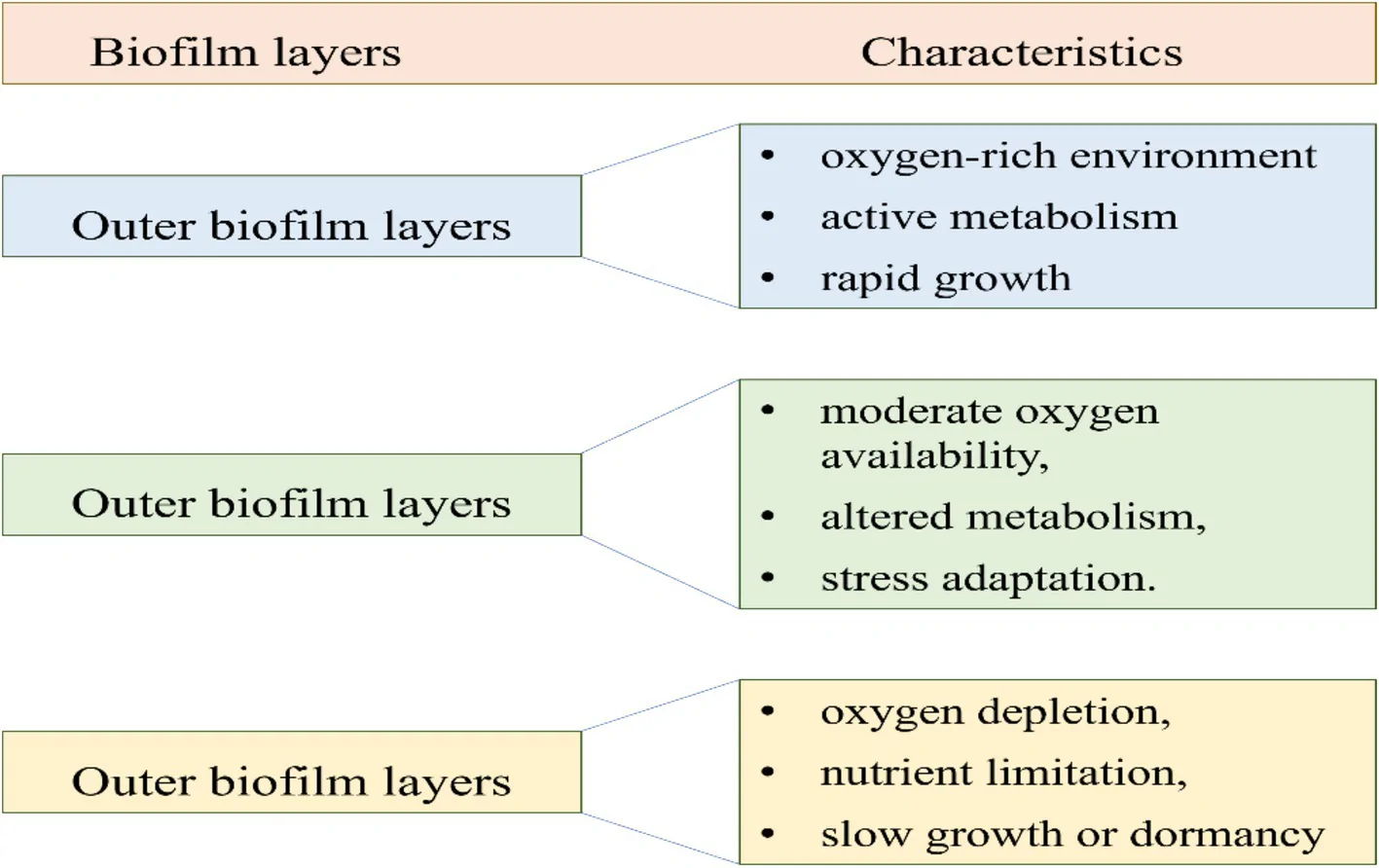
Spatial and physiological heterogeneity within a mature biofilm. A conceptual representation of a mature biofilm with spatial variations in oxygen, nutrients, penetration of antimicrobial agents, and metabolic activity. Actively growing cells exist on the surface, in between intermediate stress-adapted cells, and deeper regions contain dormant persister cells. These spatially differentiated microenvironments contribute to heterogeneous antimicrobial susceptibility and survival.

### 4.2 Persister formation and survival states

Persistence in biofilm is a result of physiological and regulatory processes that give rise to subpopulations that are able to survive exposure to antimicrobial agents. The formation of persisters has been linked to metabolic downshifts, decreased ATP levels, toxin–antitoxin systems, stringent-response pathways, translationally suppressive systems, and other stress-response mechanisms that lower the metabolic processes targeted by antimicrobial drugs. Not every part of the biofilm can necessarily have these mechanisms, and the nutrient, oxygen, redox and antimicrobial gradient that exists in space can promote enrichment of persisters in particular areas of the biofilm. It is crucial to distinguish between dormancy or slow growth and persister formation, which is a survival phenotype that is related to a specific physiological and regulatory state and by which cells are heterogeneously distributed across different contexts (Ciofu et al., 2022; Balaban et al., 2019).

The main evolutionary importance of persistence is that it allows viable bacteria to remain in the population when there is high antimicrobial selection pressure. Persisters can regrow and repopulate the biofilm after the removal or reduction of antimicrobial pressure resulting in recurrence and prolonged infection. But persistence alone does not equal resistance to antimicrobials and should not be regarded as a necessary step in the development of resistance. Preserving viable cells during antimicrobial exposure, persistence can affect subsequent evolutionary trajectories by adding more opportunities for mutation, selection, genetic exchange and ecological adaptation to occur (Balaban et al., 2019; Harms et al., 2016). The ability of these processes to lead to stable resistance is influenced by population size, genetic diversity, level and/or length of antimicrobial exposure, ecology, and accessibility of resistance determinants. Therefore, persister formation can best be thought of as a mechanism that helps to survive when selected for, but may not necessarily lead to resistance evolution.

### 4.3 EPS-mediated adaptation

Biofilm architecture is highly heterogeneous and varies according to microbial species, environmental conditions, substrate characteristics, and biofilm maturity. The EPS matrix forms a hydrated three-dimensional scaffold composed mainly of polysaccharides, proteins, extracellular DNA, and lipids, which provides structural stability and creates distinct microenvironments within the biofilm. Mature biofilms generally exhibit dimensions at the micrometre scale, whereas nanoscale measurements typically describe specific matrix components or localized structural features rather than the overall biofilm structure (Chen et al., 2026; Pattem et al., 2018). Although the EPS matrix can influence antimicrobial transport through diffusion limitation, adsorption, chemical interactions, and enzymatic modification, it should not be considered an absolute barrier to antimicrobial penetration. Many antimicrobial agents can reach biofilm-associated cells, yet substantial tolerance may persist due to physiological heterogeneity, reduced metabolic activity, stress adaptation, and persister-cell formation. Therefore, the contribution of EPS to antimicrobial survival is dependent on the antimicrobial compound, microbial species, matrix composition, and local biofilm microenvironment rather than representing a universal mechanism of protection (Stewart and Franklin, 2008). EPS matrix of *P. aeruginosa* consists mostly of alginate, other polysaccharides including Pel and Psl are also found (Qureshi et al., 2026). Alginate ensures structural integrity and protection of the biofilm. It has been shown that the *P. aeruginosa* biofilms have the ability to impede antibiotic penetration of ciprofloxacin through the alginate matrix. This membrane diffusion barrier provides that the antibiotic diffuses to the deeper parts of the biofilm only to sub-lethal levels so as to leave the bacteria alive and surviving. Psl is a sequence of repeating units of monosaccharides such as L-rhamnose, D-mannose and D-glucose (Byrd et al., 2009). Pel, a product of *pel*ABCDEFG gene cluster, shields the *P. aeruginosa* against gentamicin and tobramycin. It is composed of 1–4 glycosidic bond between two sugar molecules such as N-actetylglucosamine and N-acetylgalactosamine (Jennings et al., 2015; Franklin et al., 2011). At physiological pH, Pel is positively charged and amino-glycoside-resistant (Colvin et al., 2011). Resistance of Pel is developed through an interaction with eDNA. eDNA that can chelate cationic antibiotics making them inactive. PelA enzyme has a hydrolase and a deacetylase domain. The production of Pel is produced by the deacetylase domain and the degradation of the Pel polysaccharide is produced by the hydrolase domain. Thus, aminoglycoside resistance property is increased when there is a mutation in the hydrolase region and not in the case of the deacetylase region (Jennings et al., 2015). eDNA in different biofilm-producing microorganisms are reported in the literature like the murine lung model for *P. aeruginosa* and chinchilla middle ear for Haemophilus influenza (Qureshi et al., 2026; Sauer et al., 2002). DNABII family proteins (histone-like proteins and integration host factor) interact with eDNA and provide them stable structures. These proteins are placed on the vertices of eDNA those accelerate the affinity for Holliday junction DNA (HJ DNA) and stabilise the biofilm matrix (Hall and Mah, 2017). *Campylobacter jejuni* take eDNA from a culture medium or free environment and transform their planktonic cell into biofilm-producing cells. Different antibiotic resistance genes like *aphA3*, *cat* present in their chromosomal DNA to resist the action of kanamycin and chloramphenicol, respectively, (Qureshi et al., 2026). *E. coli* builds biofilm matrix consisting of phosphoethanolamine (pEtN)-altered cellulose as well as amyloid protein fibres. pEtN transferase effects the addition of pEtN onto cellulose exopolysaccharide (Anderson et al., 2021). Polymyxins attack the outer membrane lipopolysaccharides of gram-negative bacteria. The antimicrobial drugs (AMDs) contain the positively charged cyclic polypeptide, the major component, and its activity is the same as that the cationic detergents. Polymyxin molecules react with the negatively charged phosphate groups of lipopolysaccharides, and the neutralisation of the membrane charge occurs, as well as the intracellular and external components permeability varies. The very principle of polymyxin resistance is the inhibition of penetration pathway of the antibiotic into the cell. This channel is closed by the modification of lipid A with phosphoethanolamine, which is done by phosphoethanolamine transferase. This enzyme has a gene that is coded in the chromosome. *Mcr-1* gene has recently been identified on plasmids. The development of biofilm resistance is associated with the mutations in the phosphoethanolamine transferase genes (Schumann et al., 2024). Similarly, enzymes and structural proteins contribute to matrix organization and may influence antimicrobial activity. β-lactamases are capable of degrading beta-lactam antibiotics, and changes in the enzyme can also alter the structure of other antibiotics, which will make them useless. This enzyme barrier provides another level of protection against the treatment process of antibiotics. β-lactamase is an enzyme secreted by *P. aeruginosa* in the biofilm matrix (Singh et al., 2025; Sauer et al., 2002). This enzyme resists β-lactam antibiotics. Aminoglycoside modifying enzymes (AMEs) are key factors to develop resistance against aminoglycoside antimicrobials. It helps in the modification of aminoglycoside antibiotics through phosphorylation, acetylation, or adenylation, reducing their binding affinity to bacterial ribosomes. Aminoglycoside phosphotransferases (APHs), aminoglycoside acetyltransferases (AACs), aminoglycoside nucleotidyltransferases (ANTs) are the enzymes under AME. These enzymes are upregulated in biofilms, contributing to aminoglycoside resistance (Qureshi et al., 2026; Alhede et al., 2011).

### 4.4 Stress responses and regulatory networks

Biofilm survival is controlled by interconnected regulatory systems that allow bacterial cells to respond to nutrient limitation, oxidative stress, antimicrobial exposure, and other environmental changes. Rather than functioning as independent pathways, stress-response systems, quorum sensing, c-di-GMP signalling, two-component systems, small regulatory RNAs, and transcriptional regulators interact to coordinate biofilm development and adaptation. Stress-associated physiological adaptation can interact with regulatory signalling to alter bacterial survival under biofilm conditions (Figure 4a).

**Figure 4:**
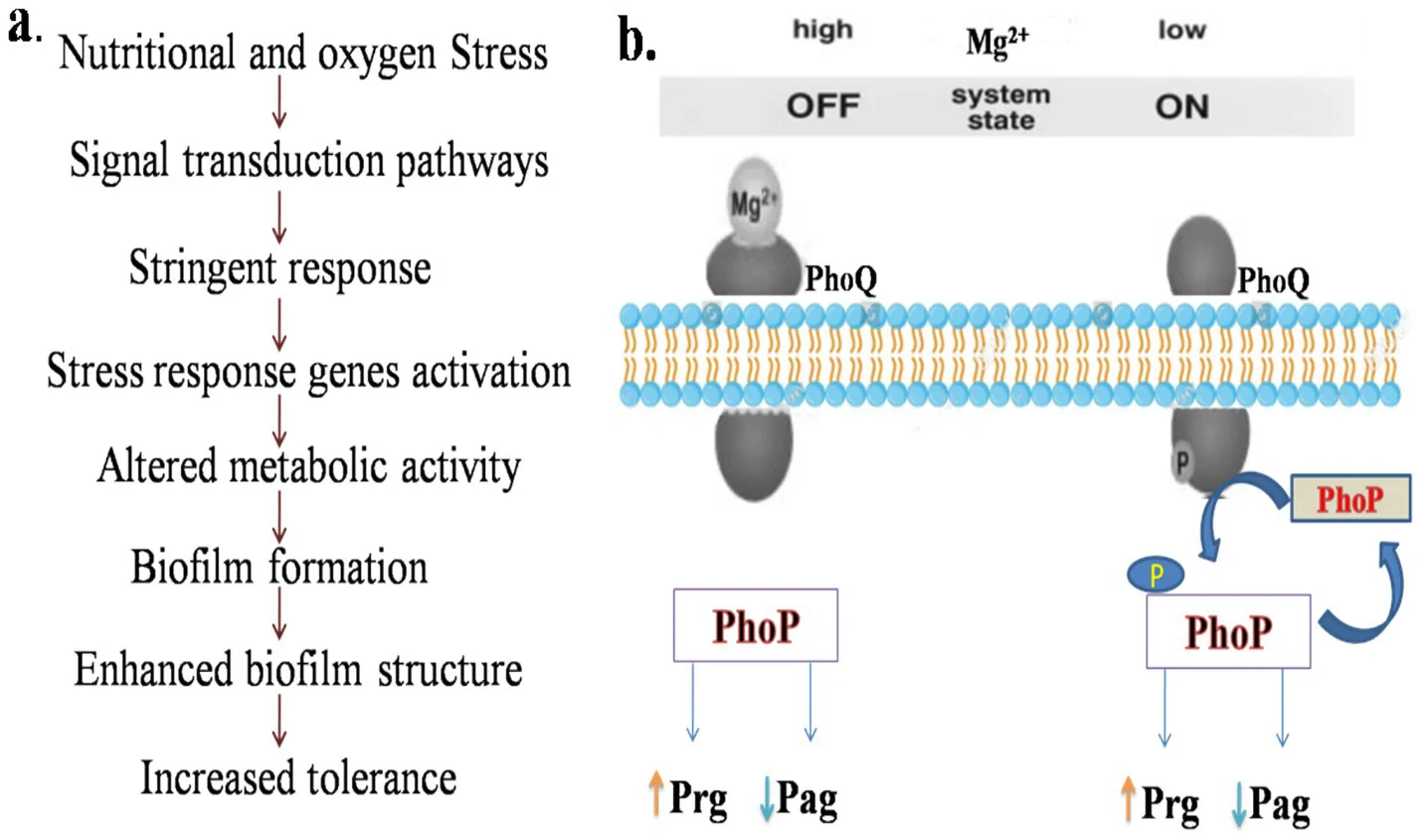
Development of biofilm tolerance in response to nutritional and oxygen stress conditions **(a)**. Model for the regulation of the *Salmonella* PhoP/PhoQ Two-Component System by Mg^2+^ **(b)**. When exposed to millimolar doses of Mg^2+^, PhoQ takes on a shape that enhances its phosphatase activity or decreases its kinase activity, or possibly both. Because of this, phospho-PhoP-reducing expression of *Pho*P-activated (Pag) genes decreases. The PhoP-repressed (Prg) genes are actively involved in transcription when this status is off. A higher quantity of phospho-PhoP results from a conformation of PhoQ that either enhances its kinase activity or suppresses its phosphatase activity, or both, in a low-magnesium environment. A positive feedback loop is established when this activates Pag genes, such as phoPQ, which in turn increases the response. At this condition, the Prg genes are not actively involved in gene transcription. The ability to live inside macrophages and resistance to antimicrobial peptides are virulence-related characteristics that bacteria acquire when they enter the ON state.

#### 4.4.1 Stringent and general stress responses

Nutrient limitation and oxygen deprivation can activate stress-associated pathways that reduce growth-associated processes and enhance survival functions. A change in concentration of nutrients and oxygen affect bacterial biofilm growth (Azeem et al., 2025). Cells within the oxygen-limited biofilm layer exhibit low metabolic activity and remain in a stationary-phase like state. This slow growth of the bacteria is considered for the antimicrobial tolerance, as most antimicrobials target the fast-growing microbes (Hall and Mah, 2017). Nutritional stress condition activates a stringent response system, that allows the bacteria to survive in environmental stress condition as shown in Figure 4a. This is achieved by the production of alarmone guanosine 5′ -(tri) diphosphate 3′ -diphosphate by *SpoT,* resulting in the downregulation of growth-associated genes and the upregulation of survival-associated genes (Chau et al., 2021). This response provides more tolerance to *Escherichia coli* against antibiotics like penicillin. A mutant condition of *E. coli*, which was disabled to produce alarmone guanosine 5′ -(tri) diphosphate 3′ -diphosphate was found more susceptible for penicillin (Li et al., 2016). This is produced by *relA* gene. In the presence of antimicrobial agents, phospholipid biosynthesis may be hampered. This could be overcome by an accumulation of phospholipid biosynthetic enzyme, i.e., *sn*-glycerol-3-phosphate acyltransferase (PlsB). Alarmone guanosine 5′ -(tri) diphosphate 3′ -diphosphate provides an opportunity to elevate the accumulation of PlsB. A stringent response system also provides antimicrobial tolerance to *Mycobacterium tuberculosis, B. subtilis* and *Streptomyces coelicolor.* Resistance development against polymyxin B, trimethoprim, and gentamicin were reported previously by this response system (Hall and Mah, 2017). Phosphate and amino acid deprivation provide another tolerance mechanism against ofloxacin and ampicillin to *E. coli,* respectively (Li et al., 2016). The nutritional limitations also contribute in higher magnitude against different antimicrobials as shown in Figure 4b. During low levels of Mg^2+^, PhoPQ (two-component system) in *Salmonella* gets activated. PhoQ acts as sensor kinase, which senses the limitation and activates response regulator, PhoP (Omelaniuk et al., 2026). This two-component system (TCS) now up-regulates different genes, those heighten the adaptation level under stress condition and protect the bacteria from CAP (cationic antimicrobial peptides) family antibiotics like polymyxin B and colistin. Mutation in this system has been observed to increase susceptibility to those antimicrobials (Li et al., 2016). Another two-component system comprising of *pag*P and *pmr*AB works along with *Pho*PQ for the addition of palmitate and 4-amino-arabinose to lipid A, respectively. Resulting in the occurrence of positive charge in the surface area is noted, that oppose entry of CAP family antimicrobials. A low level of Fe^3+^ activates pmrAB that gets conjugated with PhoPQ and regulates phosphoethanolamine transferase protecting the microorganism from CAP family antimicrobials. *P. aeruginosa, Klebsiella pneumoniae*, and *Yersinia pestis* also have this type of defence system against antibiotics (Cheng et al., 2010). RpoS considered to be a key component is found to be responsible for their adaptation in an alternative environment. RpoS is a sigma factor present in gram-negative bacteria. Mutant RpoS shows impaired biofilm development in *E. coli* but well-upholstered biofilm in *P. aeruginosa* (Horna et al., 2018).

#### 4.4.2 Oxidative stress responses

Reactive oxygen species (ROS) have been proposed as contributors to antimicrobial-mediated killing, although the magnitude and universality of this mechanism remain debated, usually depends on the organism, antimicrobial, metabolic state, and experimental conditions. In contrast, oxidative stress responses and antioxidant defense systems are well-established components of bacterial adaptation during biofilm development and antimicrobial exposure. Reduced nicotinamide adenine dinucleotide (NADH), generated during central metabolism including the TCA cycle, donates electrons to the electron transport system (ETS), contributing to respiratory electron flow and reactive oxygen species generation. Superoxide radicals convert iron (II) to iron (III) of iron–sulphur cluster, thus these free radicals can cause cell death. It is also known that production of ROS is increased during antibiotic treatment (Fu et al., 2026). Managing antibiotic-induced ROS in biofilms could be one of the many methods that biofilm cells use to acquire a high level of antibiotic tolerance. *P. aeruginosa* biofilms, for example, had elevated ROS levels after ciprofloxacin treatment, even at sub-inhibitory antibiotic doses, and increased ROS levels linked with lower cellular viability. Given that biofilms created by a katA mutant produced more ROS after ciprofloxacin treatment and were also more sensitive to ciprofloxacin than wild-type biofilms, this antibiotic-induced ROS is partially detoxified by the KatA catalase. Adding thiourea, a scavenger of hydroxyl radicals, to ciprofloxacin therapy not only lowered the amount of ROS produced, but also increased the survivability of the *P. aeruginosa* biofilm (Jensen et al., 2014). This suggests the reduced tolerance of katA mutant biofilms to ciprofloxacin. Another mechanism that contributes to *P. aeruginosa* biofilm tolerance to antibiotics is the stringent response, which reduces pro-oxidant molecules while upregulating antioxidant mechanisms like KatA. Tobramycin or ciprofloxacin exposure increased intracellular ROS in *Burkholderia cepacia* complex biofilms. When some antioxidants, such as glutathione, were added at the same time as antibiotic treatment, biofilm cell viability was dramatically enhanced. In support of the concept that ROS detoxification is critical for biofilm tolerance, Van Acker et al. (2016) found that tobramycin and ciprofloxacin treatment reduced the survival of ∆*kat*B *B. cepacia* complex biofilms by 40 and 30 times, respectively, compared to wild type (Van Acker et al., 2016).

#### 4.4.3 Quorum sensing

Quorum sensing enables bacteria to coordinate gene expression in response to population density through diffusible signalling molecules. In biofilms, quorum-sensing systems can influence attachment, matrix production, maturation, dispersal, virulence-associated traits, and antimicrobial susceptibility. Quorum sensing provides a population-dependent regulatory mechanism through which bacterial cells coordinate collective behaviours according to cell density (Figure 5). Evidence from *P. aeruginosa*, *S. aureus*, and *Enterococcus faecalis* indicates that disruption of specific quorum-sensing pathways can alter biofilm-associated antimicrobial responses. Biofilms formed by the *P. aeruginosa* ∆*las*R ∆*rhl*R strain that lacks quorum sensing were considerably more sensitive to tobramycin than biofilms formed by their wild-type counterparts. It has been reported that in *P. aeruginosa*, some colistin-tolerant subpopulations in biofilms are linked with the quorum sensing (Omelaniuk et al., 2026). A quorum-sensing-deficient ∆*agr*D mutant in *S. aureus* showed a biofilm-specific reduction in rifampin resistance compared to wild-type controls. Moreover, the quorum-regulated *gel*E protease and the *fsr* quorum-sensing system were necessary for biofilm resistance to linezolid, daptomycin and gentamicin in *E. faecalis* (Dale et al., 2015). Inhibition of the quorum-sensing receptor, TraP by hamamelitannin significantly increases the antimicrobial efficacy of various antibiotics, i.e., cephalosporins, vancomycin, daptomycin, linezolid, tobramycin, and fusidic acid in pre-established *S. aureus* biofilms by reducing peptidoglycan synthesis, cell wall thickness, and matrix eDNA content (Qureshi et al., 2026; Zhou et al., 2020).

**Figure 5:**
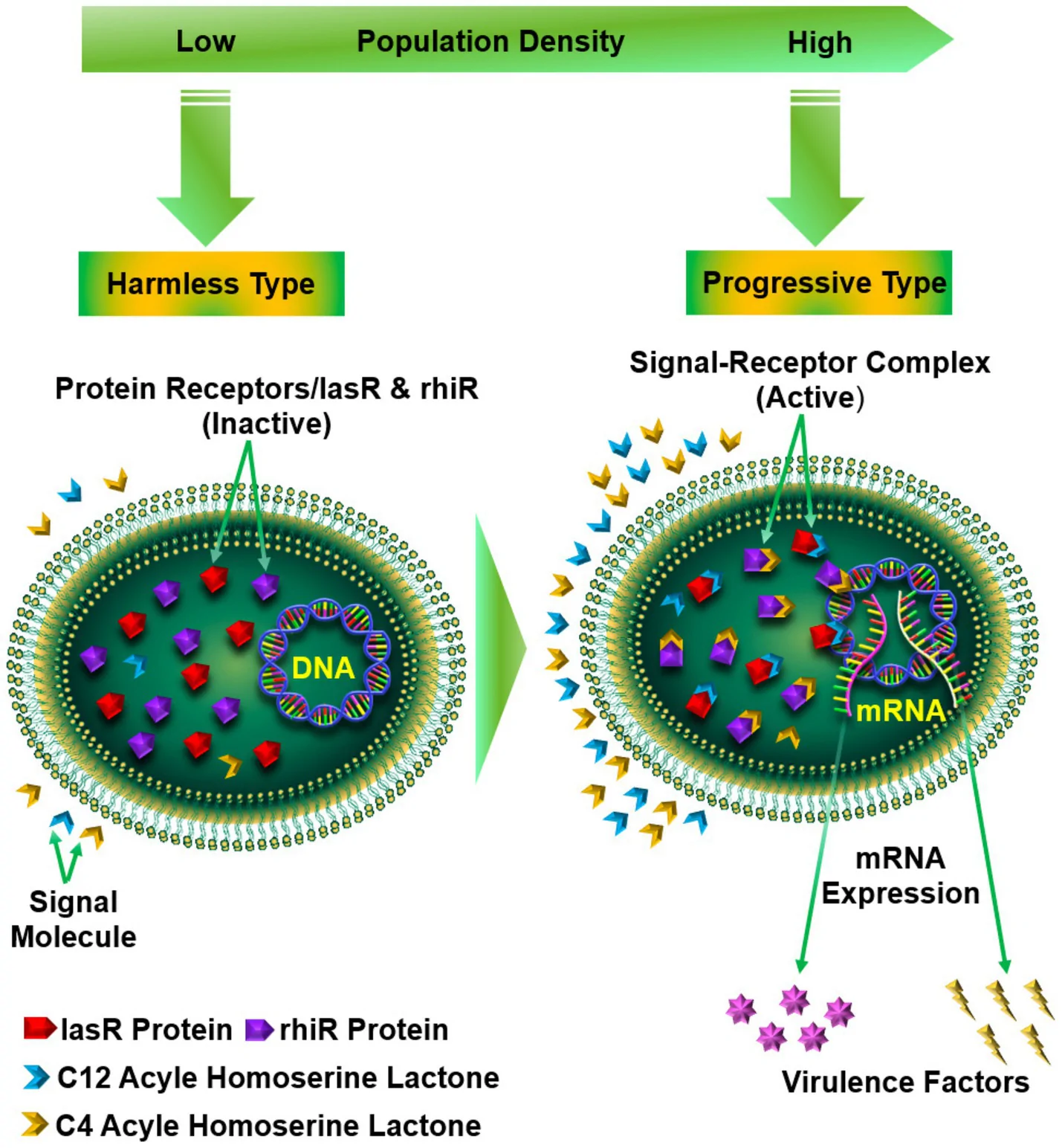
Quorum-sensing regulation according to bacterial population density. Conceptual representation of quorum-sensing signalling under low- and high-cell-density conditions. At low cell density, signal-molecule concentrations remain below the threshold required for coordinated activation of the genes regulated by the quorum-sensing system. With the increase in cell density, the accumulation of signal molecules leads to an increased activation of the receptor, and thus coordinated expression of genes that contribute to community-associated behaviours such as the development of a biofilm, matrix production, virulence and dispersal. The actual regulatory effects are species-specific and depend on the specific quorum-sensing system of the bacteria.

#### 4.4.4 C-di-GMP signalling

The cyclic di-guanosine monophosphate (c-di-GMP), an intracellular second messenger signalling molecule plays an important role in the regulation of behaviour, growth phenotypes, motility, virulence factor production and transition of lifestyle between motile to sessile state in many bacterial species. This c-di-GMP was primarily reported in *Acetobacter xylinus* as an allosterical activator for cellulose synthase (Qureshi et al., 2026). It is synthesized from the two GTP molecules with the help of the enzyme, di-guanylate cyclase that is characterized by the presence of a highly conserved active glycine–glycine–aspartate–glutamate–phenylalanine (GGDEF) domain. Phosphodiesterase degrades this c-di-GMP into pGpG or GMP molecule. Digyanylate cyclase and Phosphodiesterase regulate the intracellular c-di-GMP level in a cencertive fashion (Sauer et al., 2002). Several studies reported about the association between c-di-GMP and biofilm formation in many clinically important bacteria such as *P. aeruginosa*, *E. coli*, *S. enterica* and serovar *Typhimurium* (Pietsch et al., 2020). On average 75–110 pmol of c-di-GMP per mg was estimated in the case of *P. aeruginosa* biofilms, whereas it was less than 30 pmol per mg in their planktonic counterparts. Bacteria execute this lifestyle transition by modulating the localization of cell surface protein, transcriptional activation, various protein–protein interactions as well as DNA binding properties and hence, regulates several effector proteins, which can bind to the c-di-GMP (Sharma et al., 2026). Binding of an inner membrane effector protein LapD with the intracellular c-di-GMP initiates the biofilm formation by facilitating the transport of LapA (a biofilm adhesion protein) to the cell surface (Yasbolaghi Sharahi et al., 2026). Several other studies also supported the role of LapA with the biofilm formation, where inhibition of LapA in presence of RapAPDE, diminished the biofilm formation (Yasbolaghi Sharahi et al., 2026). Here, RapAPDE was suggested to have a role in the reduction of the intracellular level of c-di-GMP (Qureshi et al., 2026). In the case of *E. coli*, a protein–protein interaction was reported, where YcgR protein regulates the intracellular level of c-di-GMP and interacts with the components of the flagellar motor during motile to sessile lifestyle transition of bacteria. In *V. cholerae*, a sensor kinase/response regulator pair named VieS/VieA reduced biofilm formation by decreasing the intracellular concentration of c-di-GMP (Conner et al., 2017). VieS is a hybrid sensor kinase, whereas VieA is its response regulator in *V. cholerae*. VieS has a role in sensing host-specific signals and changes the gene expression pattern by phosphorylating VieA. *VieA* mutant of *V. cholerae* reportedly showed decreased biofilm formation. VieS phosphorylates the response regulator VieA phosphodiesterase and thereby results in the degradation of c-di-GMP. The association between the reduced intracellular level of c-di-GMP with the inhibition of *vps* genes being responsible for the synthesis of EPS as well as the formation of biofilm was reported (Conner et al., 2017; Li et al., 2016). Elevated levels of this c-di-GMP contribute to antimicrobial resistance of *P. aeruginosa.* The two-component hybrid SagS and its modulation of the intracellular concentration of c-di-GMP through an unidentified diguanylate cyclase are tightly linked to the expression of BrlR transcription factor (Azeem et al., 2025). *Brl*R gene expression could be increased in planktonic and biofilm cells in clinical *P. aeruginosa* isolates from cystic fibrosis (CF) patients compared to the laboratory strain PAO1 *in vitro*, and this increased expression appeared to contribute to the comparatively high degree of tobramycin tolerance reported in CF isolates (Qureshi et al., 2026). PvrR has the ability to degrade c-di-GMP, a crucial second messenger. Biofilm generated by *P. aeruginosa gac*S cells gave rise to phenotypically stable Small Colony Varients (SCVs) that were tolerant to antimicrobials. The formation of SCVs in *P. aeruginosa* biofilms may be a significant determinant of biofilm recalcitrance in these Gram-negative nosocomial pathogens.

#### 4.4.5 Small regulatory RNAs and transcriptional regulation

Small noncoding RNAs (sRNAs) also play a pivotal role in biofilm regulation in several bacterial species. These sRNAs regulate biofilm formation by binding with several proteins or through base pairing with the other RNA (Li et al., 2026; Ayenew et al., 2026). CsrB, a sRNA and CsrA, a RNA binding protein do have a significant impact on the formation as well as dispersion of biofilm in *E. coli* (Yasbolaghi Sharahi et al., 2026). In *P. aeruginosa*, GAC signal system is a prominent example of sRNA mediated biofilm regulation. GacS is a sensor kinase located in the transmembrane region and GacA is its cognate regulator. GacS regulates this GAC cascade by phosphorylating GacA (Li et al., 2026). After the phosphorylation, GacA potentiates the synthesis of two sRNA namely RsmY and RsmZ. RsmA is a regulatory protein that can bind with the RsmY and RsmZ. RsmA itself can function as a transcriptional repressor and thereby causes the reduction of biofilm formation. The binding of RsmA with the RsmY and RsmZ inhibits the repressor activity of RsmA and induces switching the planktonic bacteria into its biofilm structure (Ayenew et al., 2026). Another novel regulatory sRNA called SrbA, has been reported in *P. aeruginosa* where it strongly regulates biofilm formation. Overexpression of SrbA gene was reported in *P. aeruginosa* strain PA14 after the biofilm culture (Taylor et al., 2017; Chambers and Sauer, 2013). Evidence is mounting that regulatory RNAs play a role in antibiotic resistance, making these molecules interesting targets for antimicrobial treatment. By base-pairing with mRNAs encoding activities critical for resistance such as metabolic enzymes, drug efflux pumps, or transport proteins, trans-encoded short RNAs (sRNAs) influence antibiotic tolerance. Antibiotics cause bacteria to modify their sRNA repertoire significantly. Each antibiotic produces a distinct sRNA profile, which may have downstream effects that aid in overcoming the antibiotic challenge. Hfq increases the creation of the sRNA/target RNA duplex in Gram-negative bacteria, influencing the target RNA’s translation, degradation, or transcription. Because Hfq and CsrA are required for the action of many cognate sRNAs, inhibiting them has been demonstrated to down-regulate sRNA networks that govern a variety of virulence-related activities, making bacteria not only non-infectious but also more vulnerable to drugs (Taylor et al., 2017). Table 2 shows several trans encoded sRNA, which are associated with the antimicrobial resistance.

**Table 2:** Trans-encoded sRNA contributing to antimicrobial resistance in different microorganisms and their mechanism of resistance.

sRNA	Microorganisms	Antimicrobial resistance	Mechanism	References
SroC	*Salmonella* sp.	Polymyxin B	SroC contributes to resistance by downregulation of sRNA *Mgr*R	Acuña et al. (2016)
SprX (RsaOR)	*S. aureus*	Glycopeptides	Deletion mutation increases the resistance. SprX acts by repression of *spoVG*	Yang et al. (2025)
SdsR (RyeB)	*E. coli*	Ampicillin	Ampicillin promotes mutations through repression of *mutS* by SdsR. Mutations may confer resistance	Gutierrez et al. (2013)
MicF	*E. coli*, *Salmonella* sp.	Cephalosporins, norfloxacin	Overexpression increases the resistance through repression of *omp*F	Felden and Cattoir (2018)
GlmY, GlmZ	*E. coli*, *Salmonella sp.*	GlmS inhibitors (Bacilysin, Nva-FMDP)	Confer resistance via overproduction of *Glm*S	Choi et al. (2018), Khan et al. (2016)
GcvB	*E. coli*	D-cycloserine	GcvB provides resistance by repression of *cycA*, which is required for drug uptake	Updegrove et al. (2016)
DsrA	*E. coli*	Oxacillin, erythromycin, novobiocin	Overexpression provides resistance through upregulation of efflux pump *Mdt*EF via *Rpo*S	Dersch et al. (2017), Updegrove et al. (2016)

Transcriptional regulators are proteins that regulate gene expression which are important in helping bacteria to adjust to stressful environments, such as presence of antimicrobial agents. These regulators coordinate the intricate gene expression programs which form the basis of biofilm resistance by regulating the activity of RNA polymerase (RNAP) and other components of the transcriptional machinery. *P. aeruginosa* LasR and RhlR are quorum sensing (QS) regulators, which regulate the expression of genes related to biofilm maturation, virulence and resistant genes (Pang et al., 2019). These regulators attach to the particular promoter areas when the autoinducers are present leading to the activation of the target genes in the synthesis of rhamnolipids and other extracellular polysaccharides.

TCS acts as a key factor to regulate the transcription process. TCS receives various signals from the surrounding environment and regulates the level of gene expression. In *P. aeruginosa* out of 130 TCSs, 60 TCSs are engaged in antimicrobial resistance mechanisms (Pang et al., 2019). RocS2-RocA is an example of TCS, that upregulates the production of biofilm by down-regulating the expression of *mex*AB-*opr*M. Another TCS is PmrAB, encoded by *pmr*ABC operon. This operon produces three different proteins: PmrA (regulatory protein), PmrB (cytoplasmic membrane bound sensor kinase), and PmrC (PEtN transferase). PmrB gets activated by a high level of iron and aluminium concentration. PmrA is activated by the kinase activity of PmrB. Besides, PmrA activates transcription of the *pbg*P operon for Ara4N modification. Modification of the PmrAB TCS is the most prevalent polymyxin resistance mechanism. PmrA can be activated by mutations in the *pmr*A or *pmr*B gene, that causes upregulation of PmrC and *arn*BCADTEF*-pmr*E operon. The encoded enzymes in this operon are responsible for the production and transfer of phosphoethanolamine and 4-deoxyaminoarabinose to lipid A (Sato et al., 2018). Under low-Mg^2+^ conditions, activation of the PhoP/PhoQ two-component system promotes transcription of *pmr*D. *Pmr*D subsequently enhances *Pmr*A activity by protecting phosphorylated PmrA from dephosphorylation by PmrB, thereby enabling expression of *Pmr*A-regulated genes involved in lipid A modification and antimicrobial peptide resistance (Rubin et al., 2015). Seven genes are present in *pbg*P operon which is also called *pmr*F locus (Pang et al., 2019). Acceleration in PmrA phosphorylation facilitates the expression of *pbg*P operon, and consequently develops resistance against colistin of *E. coli* ST59 (Sato et al., 2018). Deletion of 12 nucleotides in PmrB also develops resistance against colistin in *Salmonella enterica* ST45. Due to the down-regulation of expression of *pbg*P operon and *Pmr*C by deletion mutation in *Pmr*AB, susceptibility to colistin also decreases. *Pmr*AB and *pbg*P are regulated by CrrAB TCS. This TCS encodes three proteins: CrrA (regulatory protein), CrrB (sensor kinase), and CrrC (modulator). *Crr*B mutation facilitates phosphoethanolamine (PEtN) and 4-amino-4-deoxy-L-arabinose (Ara4N) modifications mediated by PmrC and the pbgP-associated pathway, respectively. Mutations at position 84 of HAMP (histidine kinases, adenylyl cyclases, methyl binding proteins, phosphatases) domain and, at positions 141, 151, 183 of histidine kinase A domain of CrrB increase the resistance against colistin in *K. pneumoniae*. PmrAB and CrrAB are connected through CrrC. Susceptibility to polymyxin gets reduced by a mutation in the CrrB through up-regulation of expression of H239_3064 efflux pump (Sato et al., 2018).

PhoP (regulatory protein) connected with PhoQ (membrane bound sensor kinase) by PmrD, produces a system called PhoPQ (Choi et al., 2016). PmrD is not uniformly distributed among *Enterobacteriaceae*, and its regulatory role differs among species. Single amino acid substitutions at positions 26 and 46 are responsible for loss of function of PhoQ in *K. pneumoniae* and that is considered as one of the key factors for colistin resistance. Mutation at position 191 of PhoP is responsible for disruption of *α*-helix, resulting in increased resistance to colistin (Pang et al., 2019). DedA, is a membrane-bound protein that acts as a proton motive force-dependent efflux pump. This protein exerts its effect through PhoPQ system to develop polymyxin resistance. *Enterobacter cloacae* show polymyxin susceptibility at the time of Tn5 mediated insertion mutation in *ded*A gene. Besides, another protein-coding gene that develops resistance against polymyxin is *ecr*, which activates *pbg*P through PhoPQ. The *ecr* gene is responsible for developing resistance against colistin in *Enterobacter mori* A6008. *mgrB* down-regulates the function of PhoPQ by blocking the kinase activity of PhoQ in *K. pneumonia.* A representative example is provided by the PhoP/PhoQ two-component system of *Salmonella*, which responds to environmental magnesium availability and regulates genes associated with adaptation and virulence (Figure 4b). Insertion and deletion mutations could prevent the function of *mgrB*. Reports reveal that, inactivated *mgr*B containing bacterial cell when treated with polymyxin, hardens the outer capsule layer (Qureshi et al., 2026; Choi et al., 2016).

In *P. aeruginosa*, BrlR (biofilm resistance locus regulator) is a well-studied MerR-like transcriptional activator of biofilm-specific antibiotic tolerance. The discovery that the BrlR protein is present in biofilms but not in the planktonic proteome piqued researchers’ interest in the protein. The *brl*R transcript was likewise expressed preferentially in biofilms. The isogenic *brl*R deletion mutant was as vulnerable to tobramycin when growing planktonically as wild-type *P. aeruginosa*, however, biofilms generated by the *brl*R under the flowing phases were roughly 4-fold less resistant to tobramycin than wild-type biofilms (Liao and Sauer, 2012).

*AgrA*, a part of the accessory gene regulator (agr) system in *S. aureus* controls the expression of virulence factors and biofilm dispersal. In *S. aureus*, AIP is detected by the sensor kinase AgrC, which phosphorylates AgrA, and the phosphorylated AgrA subsequently regulates the transcription of *agr*-associated genes to regulate antibiotic resistance. The expression of genes that encode the production of alginate which is a major element in the biofilm matrix is regulated by *Alg*U. When the environment is stressed, *Alg*U is activated which causes the transcription of genes that produce alginate (Sauer et al., 2002). Alginate synthesis increases biofilm mechanical properties and antimicrobial resistance.

### 4.5 Efflux systems and adaptive susceptibility

Efflux systems are a diverse class of transport systems, which may affect antimicrobial susceptibility, bacterial physiology and adaptation in biofilms. The biological effects will vary based on the type of efflux pump, the species of bacteria, the presence of regulatory factors, and the conditions of the exposure to antimicrobial agents. Hence, in some cases, efflux activity is not synonymous to antimicrobial resistance. Intrinsic or acquired resistance can be achieved by constitutive overexpression or genetic modification of specific efflux systems, which help to keep intracellular concentrations of antimicrobial compounds low. In fact, in *P. aeruginosa* it has been found that many multidrug efflux systems are over-expressed and are linked to a decrease in susceptibility to several classes of antimicrobials, such as *Mex*AB-*Opr*M, in Gram-negative bacteria, *Acr*AB-*To*lC and *Nor*A in *S. aureus*. But efflux activation can also take place as a transient physiological reaction to environmental stress. Efflux may be transiently induced under certain circumstances like by antimicrobial exposure, by oxidative stress, or by changes in the environment linked to biofilm formation, which decreases intracellular antimicrobial accumulation and may lead to an adaptive tolerance without the emergence of a stable resistance phenotype. The distinct physiological and evolutionary roles of efflux systems in biofilm-associated antimicrobial responses are summarized in Table 3.

**Table 3:** Functional roles of bacterial efflux systems in biofilm-associated antimicrobial responses.

Efflux function	Biological consequence	Relationship with antimicrobial response	References
Constitutive expression or genetic overexpression	Persistent reduction of intracellular antimicrobial concentration	Intrinsic/acquired resistance	Pang et al. (2019), Hall and Mah (2017)
Stress-induced activation	Temporary reduction in antimicrobial susceptibility	Adaptive tolerance	Ciofu et al. (2022), Hall and Mah (2017)
Biofilm-associated regulation	Influences signalling, matrix production, persistence, and community behaviour	Biofilm adaptation	Pang et al. (2019), Horna et al. (2018)
Evolutionary stabilization of increased activity	Maintained survival advantage under antimicrobial pressure	Resistance evolution	Pang et al. (2019), Horna et al. (2018)

Some efflux systems are also involved in the transport of antimicrobial agents, but also in the production of the extracellular matrix, in stress adaptation, in community behaviour and in regulation of signalling molecules in biofilm associated processes. So, efflux systems can indirectly play a role in the persistence of biofilms by modifying the physiology of the bacteria other than the export of antimicrobials. Evolutionary significance is determined by whether the increase of efflux systems is transient or genetically stabilized. Efflux hyper-expression may be important for survival while an organism is exposed to the antibiotics, while mutations or regulatory changes that result in stable expression of efflux may be responsible for stable resistance phenotypes. Thus, efflux systems are not only resistance mechanisms, they also serve as an important interface between physiological adaptation and resistance evolution.

### 4.6 Horizontal gene transfer and resistance evolution

Biofilms may create ecological conditions favourable for horizontal gene transfer (HGT) because cells are maintained in close proximity and extracellular DNA can accumulate within the matrix. Transformation, conjugation, and transduction are the major mechanisms through which genetic material can move between bacteria. Conjugative transfer is particularly relevant to antimicrobial resistance because plasmids and other mobile genetic elements may carry resistance determinants. Close-contact localization and the sessile nature of the biofilm cells make them more advantageous in plasmid transferring through HGT than their planktonic counterpart (Qureshi et al., 2026). About 10^4^-fold increase in plasmid transfer frequency was reported in multi-drug-resistant *S. aureus* strain, which greatly supports the increased HGT in biofilm cells than in the planktonic form (Abe et al., 2020). Biofilm cells of *E. faecalis* relatively showed 2-fold increase in plasmid copy number as compared to their planktonic cells (Cook and Dunny, 2014). Few classes of integrons (a genetic element which contain an integration site and a gene for the enzyme integrase) often contain antibiotic resistance gene that helps in the dissemination of resistance gene among the bacteria. A 100-fold increase in integrase expression has been reported in biofilm cells in comparison with the planktonic form of the bacterium (Abe et al., 2020). These results contribute to the argument that the organization of biofilm can provide opportunities for genetic exchange. However, the process of HGT cannot be considered as an inherent, and always beneficial, characteristic of biofilms. Transfer efficiency is variable, depending on the bacterial species, compatibility of the donor and recipient strains, type of mobile genetic element, the environment, and the particular mechanism of transfer. Biofilms, therefore, should not be viewed as “hotspots” for the transfer of resistance; rather, they are ecological environments in which the transfer of resistance can occur when conditions are right. Recombination and mutation are also mechanisms of genetic diversification. In case of hypermutator phenotype in *P. aeruginosa*, an increase in the frequencies of ciprofloxacin-resistant variants in the selected biofilm models has been observed. Although these observations are important, caution must be used in interpreting them: the increase in resistant variants recovered does not necessarily indicate that all biofilms inherently increase mutation rates. It is possible that a combination of selection for pre-existing variants, changes in survival, stress-induced mutagenesis and ecological enrichment is responsible for the result observed. Thus, the evolutionary meaning of biofilms is the interplay between heterogeneous survival, selection, genetic diversification and gene exchange. Tolerant or persistent cells can sustain viable cells during antimicrobial exposure and HGT and mutation can be potential mechanisms for heritable resistance. Thus, the evolutionary course is dependent on the ecological and selective context and not only the ability to form a biofilm.

Collectively, these mechanisms demonstrate that biofilm-associated antimicrobial survival arises from interacting physiological, regulatory, structural, and evolutionary processes rather than from a single resistance mechanism. The major adaptive features and their relationships to antimicrobial survival and resistance evolution are summarized in Table 4.

**Table 4:** Biofilm heterogeneity and molecular mechanisms influencing antimicrobial survival.

Adaptive feature	Major biological components/processes	Contribution to antimicrobial survival	Relationship with resistance evolution	Representative examples	References
Spatial heterogeneity	Oxygen, nutrient, pH, and antimicrobial gradients	Generates distinct physiological states with different antimicrobial susceptibilities	Maintains surviving populations that may undergo further selection	Deep biofilm regions with reduced metabolic activity	Stewart and Franklin (2008), Sauer et al. (2002)
Metabolic heterogeneity	Growth-rate variation, altered energy metabolism, nutrient limitation	Reduces activity of antimicrobials targeting active cellular processes	May increase survival opportunities during selection pressure	Slow-growing biofilm populations	Ciofu et al. (2022), Stewart and Franklin (2008)
Persister formation	Toxin–antitoxin systems, stringent response, reduced ATP production	Enables transient survival without genetic resistance	Provides viable cells from which resistant variants may later emerge	*E. coli*, *P. aeruginosa*, *S. aureus*	Ciofu et al. (2022), Balaban et al. (2019)
EPS-mediated adaptation	Polysaccharides, proteins, extracellular DNA, lipids	Modifies antimicrobial distribution and creates protective microenvironments	May indirectly influence evolution by increasing survival duration	Alginate in *P. aeruginosa*; cellulose in *E. coli*	Flemming et al. (2023), Franklin et al. (2011), Flemming and Wingender (2010)
Stress responses	RpoS, stringent response, oxidative stress pathways	Promotes survival under antimicrobial and environmental stress	Can influence evolutionary trajectories under prolonged selection	RpoS-associated adaptation in Gram-negative bacteria	Ciofu et al. (2022), Horna et al. (2018), Jensen et al. (2014)
Quorum sensing	Autoinducers and regulatory networks	Coordinates biofilm formation, matrix production, and community behaviour	May influence population-level adaptation	Las/Rhl systems in *P. aeruginosa*	Omelaniuk et al. (2026), Papenfort and Bassler (2016)
c-di-GMP signalling	Diguanylate cyclases and phosphodiesterases	Controls transition between motile and biofilm lifestyles	Influences persistence and biofilm maturation	*P. aeruginosa*, *Vibrio cholerae*	Ciofu et al. (2022), Conner et al. (2017)
Efflux systems	AcrAB-TolC, Mex systems, NorA	Reduces intracellular antimicrobial concentration and modifies adaptation	Can contribute to resistance when genetically stabilized	MexAB-OprM in *P. aeruginosa*; NorA in *S. aureus*	Pang et al. (2019), Horna et al. (2018), Hall and Mah (2017)
Horizontal gene transfer	Transformation, conjugation, transduction	Enables acquisition of resistance determinants	Direct route to stable resistance	Plasmid-mediated resistance transfer	Abe et al. (2020), Dale et al. (2015), Cook and Dunny (2014)

## 5 Emerging therapeutic vulnerabilities and anti-biofilm strategies

Failure of antimicrobial treatment in the above-mentioned mechanisms is not solved just by increasing the dose of conventional antibiotics in the treatment of biofilms. Extracellular matrix, regulatory signalling, metabolic heterogeneity, persistence states and adaptive efflux are all modulators of antimicrobial exposure and/or cellular susceptibility and are therapeutic vulnerabilities. The therapeutic aim should therefore not just be aimed at killing the bacteria, but at interfering with, or changing, the physiology and ecology that allows them to survive and if possible, regain antimicrobial susceptibility. This is important as a decrease in biomass of biofilm does not necessarily mean killing bacteria and disruption of the matrix may only make the bacteria or aggregates viable giving them a chance to multiply, which is then followed by antimicrobial treatment, host clearance or physical removal. So, for therapeutic evaluation, there should be consideration of clinically relevant outcomes and recurrence, as well as biomass reduction and viable-cell reduction. Recent evidence suggests that it is better to consider combination of matrix-disrupting, signalling-modulating, biological or delivery-based interventions with conventional antimicrobials, instead of a single adjunctive mechanism (Qurbani et al., 2026).

### 5.1 Matrix vulnerabilities

The extracellular matrix is an exposed structural weakness of a biofilm but therapeutic targeting is better considered as matrix “sensitization” rather than eradication. Selective degradation of extracellular DNA, polysaccharides and matrix proteins may lead to loosening of the biofilm structure, thereby creating more access to antimicrobial agents and host immune effectors. Enzymatic methods involve DNase I for eDNA, dispersin B and other glycoside hydrolases for polysaccharide-based matrices, and proteases for protein-based matrices and alginate lyases for alginate-rich matrices. Sensitisation effect is species-specific, matrix and niche dependent, and matrix degradation alone has limited bactericidal activity. Matrix-targeting approaches are logical when combined in time to effective antimicrobial therapy or immune clearance/physical removal of the matrix, since complete or incomplete dispersal may allow the release of viable cells. Therefore, there is now evidence to support the “disrupt-and-kill” or “detach-and-clear” concept, rather than matrix disruption alone. Recent preclinical studies of antimicrobial peptides (AMP) and nanomaterial delivery systems also highlight the importance of synergizing structure or delivery effects with direct killing of bacteria (Qurbani et al., 2026; Makabenta et al., 2023; Park et al., 2019).

Another strategy is to inhibit matrix production instead of to deteriorate a set up matrix. EPS production is regulated by c-di-GMP, quorum sensing and other species-specific mechanisms, and disruption of these can impact the maturation of the biofilm or susceptibility during the development of the biofilm. However, the composition of the matrix varies widely among bacterial species and anatomical niches and so, universal matrix targeting is not likely to be effective across the clinically-diverse biofilms (Flemming et al., 2023).

### 5.2 Regulatory vulnerabilities

The second class of therapeutic targets are the regulatory networks that control the growth and adaptation of the biofilms. Attachment, matrix production, maturation, dispersal, metabolism and antimicrobial susceptibility are influenced by quorum sensing, c-di-GMP signalling, two-component systems, small regulatory RNAs and stress-response pathways. The targeting of these systems can impair multiple biofilm phenotypes at once, and need not directly apply a bactericidal pressure. Quorum-quenching strategies may affect either the production of the signal, the detection of the signal, or the downstream regulation in response to the signal, as well as the maturation of the biofilm, production of the matrix, and coordinated stress adaptation (Koo et al., 2017). Quorum sensing, however, should not be thought of as the universal “master switch” in biofilm resistance, as bacteria have redundant signalling systems, which play more or less important roles depending on species and environmental conditions, e.g., c-di-GMP signalling which regulates the transition from motile to sessile lifestyles and activities within the matrix. However, there are several diguanylate cyclases and phosphodiesterases that need to be inhibited, and this can be rather unpredictable, preferring selective targets of pathogen-specific signalling components. Precision gene regulation is a complementary approach; the quorum-sensing gene *lux*S was targeted by CRISPR interference to reduce the amount of biofilm produced by *E. coli*, but this is still an experimental technique and not a clinically proven treatment (Zuberi et al., 2017). Stress-response pathways could be targeted as well, however due to their strong association with critical bacterial physiology, selectivity, toxicity and compensatory responses would be concerns. More general, recent translational evaluations have pointed out that non-antibiotic methods are not necessarily ‘bacteria-proof’ because of pathway redundancy and alternative survival strategies. Therefore, regulatory interventions should be rather pathogen and context specific adjuncts and not universal anti-biofilm drugs.

One of the key conceptual strengths of regulatory targeting is that it focuses on adaptive capacity as opposed to one resistance determinant. Single-target approaches are, however, limited by pathway redundancy and compensatory regulation. Combination strategies should, therefore, be aimed at complementary regulatory or survival processes and tested experimentally for pharmacodynamic interaction and possible antagonism, instead of being taken-for-granted, as synergistic (Qurbani et al., 2026).

### 5.3 Metabolic and persistence vulnerabilities

A problem that metabolic heterogeneity poses is that many traditional antimicrobial agents are more effective against proliferating cells. Meanwhile, metabolic heterogeneity provides a chance to target metabolically reduced or persistent subpopulations. These strategies might include resensitizing the cells by inducing metabolism prior to antimicrobial exposure, or targeting molecular pathways that underpin persistence such as toxin–antitoxin systems, stringent-response signalling, ATP regulation and stress adaptation (Koo et al., 2017). The goal is not always to eliminate the persister state per se but to avoid protected physiological states being juxtaposed to antimicrobial exposure. Protected states are physiologically heterogeneous, and the entry to and exit from these states may be dependent on metabolic and stress responses. Therefore, it has been proposed that persizers can be directly killed, resuscitated in a controlled fashion while treating them with antimicrobials, or their formation can be prevented. These methods are largely preclinical and/or experimental and promising laboratory phenotypes do not necessarily translate into clinical benefit. Hence, recurrence, pharmacodynamic exposure, and host-associated infection models should be included in the translational evaluation, in addition to the static biofilm killing assays (Qurbani et al., 2026; Ciofu et al., 2022; Balaban et al., 2019).

Persistence also has implications for antimicrobial dosing since the presence of tolerant subpopulations can extend the selective exposure time. Even with the use of anti-biofilm adjunctive therapy, however, pharmacokinetic/pharmacodynamic optimization and appropriate antimicrobial exposure/timing of treatment remain important. The goal of therapy should be to decrease the number of viable cells and to prevent long selective windows that may allow for adaptive escape.

### 5.4 Efflux-associated vulnerabilities

Another potential exploitable vulnerability is the efflux system, which can have a simultaneous effect on intracellular antimicrobial concentration, biofilm physiology and, in some organisms, evolutionary stabilization of resistance. Efflux inhibition can lead to an increase in intracellular drug levels of antimicrobial substrates and re-sensitize them, depending on the pump, substrate and regulatory environment. The context dependence is exemplified by evidence of NorA-associated adaptation in *S. aureus* and biofilm-associated efflux in *P. aeruginosa*. Therefore, efflux-pump inhibitors are more likely to be used as add-on drugs. Bacteria have also been known to have overlapping efflux systems, which means that if one is inhibited, another one will take over, and some pumps may also play a role in signaling or maintaining membrane homeostasis. Non-selectivity can thus lead to restricted sensitization or to undesirable physiological responses (Pang et al., 2019; Horna et al., 2018). Use of a pathogen- or pump-specific inhibitor could be combined with an antimicrobial that is known to be affected by the system the inhibitor targets, thus increasing drug exposure inside the cell. But selectivity, toxicity, bioavailability and penetration into mature biofilms are all limitations to their clinical implementation. Any potential gain from evolution should be contingent as suppression of one efflux pathway does not eliminate selection via other pathways. Hence, the efficacy of combination needs to be demonstrated by suitable pharmacodynamic and interaction analyses.

### 5.5 Bacteriophage and nanotechnology approaches

Biological, peptide-based, and nanoscale approaches represent complementary strategies for addressing the drawbacks of traditional antibiotics. Bacteriophages can recognise specific bacterial hosts, replicate within susceptible cells and cause their lysis, and also, phage-encoded depolymerases or endolysins can potentially alter the structure of the biofilm matrix. The host specificity, resistance to phages, incomplete penetration into mature biofilms, immune neutralization, and formulation and regulatory hurdles are important limitations. A recent preclinical example was a lytic *P. aeruginosa* phage that was shown to have antibiofilm activity and a higher survival rate in a murine infection model, but for which clinical efficacy was not proven (Abdelghafar et al., 2023). Phage–antibiotic combinations are especially pertinent since the two treatments could act on distinct bacterial subpopulations or the order of treatment or the timing of delivery could impact the result (Qurbani et al., 2026). Another complementary approach is with synthetic antimicrobial peptides. PS1-series peptides inhibited planktonic growth and biofilm formation and preformed biofilms of both drug-resistant *P. aeruginosa* and *S. aureus*, and were effective in a mouse model with selected peptides (Park et al., 2019). These findings highlight a preclinical role for AMPs, and there are important barriers to translation including stability, host toxicity, pharmacokinetics, spectrum and delivery. Nanoparticles might be useful for intrinsic antimicrobial properties or for enhancing local drug delivery, matrix access and controlled release. Chitosan nanoparticles, chitosan nanoparticles loaded with carvacrol, ciprofloxacin-loaded zinc oxide (ZnO) nanoparticles, silver nanoparticles–chitosan systems, magnetic chitosan nanocomposites, and nanoparticles loaded into hydrogels are some of the recent preclinical examples (Godoy et al., 2025; Kolarijani et al., 2025; Rivera Aguayo et al., 2020; Bernal-Mercado et al., 2022). There is also recent evidence that a number of non-antibiotic modalities have advanced beyond simple *in vitro* evaluation, although the extent of their maturity for translation to the clinic is highly variable and efficacy of a given formulation should not be assumed for the whole class. Localized delivery systems are particularly relevant because it can enhance the exposure in the infection site and at the same time minimize systemic toxicity (Qurbani et al., 2026).

The major problem for nanomaterial-based therapy is thus not conceptual feasibility, but translation. Systematic assessment of the toxicity (cytotoxicity, biodistribution, persistence, immune interactions, manufacturing reproducibility and regulatory acceptability) of nanomaterials is required. Thus, the results of biomass reduction in static *in vitro* models cannot be correlated with clinical efficacy. Local exposure can be improved with controlled-release biomaterial systems, e.g., antimicrobial polymer-loaded hydrogels have achieved significant clearance of mature wound biofilms in a murine model, demonstrating the delivery architecture’s critical role in therapeutic efficacy (Makabenta et al., 2023).

### 5.6 Combination therapies and evolutionary considerations

The multifactorial nature of biofilm survival provides a rationale for combination therapy, but combinations need to be carefully chosen based on the degree of mechanistic complementarity and experimentally obtained interactions, and not on presumed synergistic properties. The degradation of the matrix may facilitate antimicrobial access, but does not necessarily eradicate metabolically dormant cells. QS inhibitors may promote disruption of the community organization without directly killing the bacteria, and efflux inhibitors may allow a higher intracellular concentration of the drug without killing the persisters (Makabenta et al., 2023; Qurbani et al., 2026). Based on these recent preclinical studies, combinations of matrix-active enzymes and antibiotics, quorum-sensing inhibitors and antimicrobials, phage and antibiotics, antimicrobial peptides and delivery systems, and nanoparticles containing antibiotics or containing the antimicrobial hydrogel are proposed. Evolutionary aspects should also be integrated in order to not only suppress growth but also to maintain the populations under selection. On the other hand, if there are interventions that reduce the population size, while others target different survival mechanisms, then opportunities for escape may be reduced, but this cannot be assumed. Combinations can also be antagonistic, that is, one treatment may interfere with the physiological condition, uptake or reproduction of the other. Hence, pharmacodynamic and interaction analysis should be used as methods to assess combinations, in addition to mechanistic matching.

Table 5 provides a summary of the main therapeutic weaknesses, the corresponding strategies and the limitations and evidence levels.

**Table 5:** Therapeutic vulnerabilities and emerging anti-biofilm strategies.

Biological vulnerability	Therapeutic strategy	Mechanism of action	Potential benefit	Major limitation	Current evidence level	References
Extracellular matrix dependence	DNase, dispersin B, alginate lyases, matrix-degrading enzymes	Disrupt EPS structure and improve antimicrobial accessibility	Can enhance antimicrobial access and weaken biofilm architecture when coupled to effective killing	Species- and niche-specific matrix composition; dispersal may release viable cells; limited activity alone	Experimental/preclinical, predominantly *in vitro* with limited *in vivo* validation	Qurbani et al. (2026), Flemming et al. (2023), Koo et al. (2017)
Biofilm regulatory signalling	Quorum-sensing inhibitors/quorum quenchers	Disrupt signal production, detection or downstream responses	May reduce maturation, matrix production, virulence and coordinated adaptation	Pathway redundancy, species specificity and environmental dependence	Experimental/preclinical, limited reports on clinical validation	Qurbani et al. (2026), Zhou et al. (2020), Papenfort and Bassler (2016)
c-di-GMP regulation	Modulation of diguanylate cyclases/phosphodiesterases	Alters motile–sessile transition and matrix-associated programmes	May inhibit biofilm development or promote dispersal	Complex, redundant signalling networks and context-dependent effects	Experimental/preclinical, target-specific outcomes	Conner et al. (2017), Koo et al. (2017)
Persistence-associated physiology	Metabolic activation, toxin–antitoxin or persistence-targeting strategies	Alters physiological states that support persister survival	May improve killing of persistent subpopulations or prevent entry into protected states	Persister heterogeneity; incomplete resensitization; limited clinical validation	Experimental/preclinical, lack of established clinical biofilm therapy	Ciofu et al. (2022), Balaban et al. (2019), Li et al. (2016)
Stress-response pathways	Stress-response inhibitors	Weakens adaptive responses to environmental and antimicrobial stress	May reduce survival under antimicrobial pressure	Pathway overlap with essential physiology; toxicity and compensatory responses	Experimental/preclinical, lack of translational validation	Omelaniuk et al. (2026), Ciofu et al. (2022)
Efflux-associated adaptation	Efflux pump inhibitors	Increase intracellular concentration of susceptible antimicrobials	May restore activity of selected antimicrobials	Pump redundancy, selectivity, toxicity and compensatory mechanisms	Experimental/preclinical, lack of established clinical efflux pump inhibitor (EPI) treatment against biofilm infections	Pang et al. (2019), Horna et al. (2018)
Membrane/cell-envelope vulnerability	Synthetic antimicrobial peptides (AMPs)	Membrane disruption and, for some peptides, interaction with matrix components	Can inhibit drug-resistant cells and biofilms; selected candidates show *in vivo* activity	Stability, host toxicity, pharmacokinetics, delivery and spectrum	Preclinical with selected *in vivo* evidence, biofilm-specific clinical evidence remains limited	Qurbani et al. (2026), Mookherjee et al. (2020), Park et al. (2019)
Phage susceptibility	Bacteriophage therapy and phage-derived enzymes	Specific bacterial lysis and/or matrix modification	Pathogen-specific killing and potential targeting of biofilm-associated populations	Host range, phage resistance, penetration, immune neutralization and regulatory barriers	Preclinical, selected phage approaches have entered early clinical development, but biofilm-specific clinical evidence remains limited	Qurbani et al. (2026), Abdelghafar et al. (2023), Kortright et al. (2019)
Delivery and matrix-access limitations	Nanoparticle and hydrogel-based systems	Increase local antimicrobial delivery, retention, penetration and/or controlled release	Can improve local exposure and support biofilm disruption; selected systems show *in vivo* efficacy	Toxicity, biodistribution, stability, manufacturing and regulatory challenges	Preclinical with multiple *in vivo* models, lack of human biofilm evidence	Qurbani et al. (2026); Makabenta et al. (2023), Bernal-Mercado et al. (2022), Baptista et al. (2018)
Precision regulatory targeting	CRISPR interference (CRISPRi)/CRISPR-based biofilm-targeting approaches	Sequence-specific suppression or disruption of biofilm-associated genes	May selectively interfere with quorum sensing or resistance-associated functions	Delivery into bacterial cells, target escape, off-target effects and biosafety/regulatory concerns	Experimental, proof-of-concept evidence, with limited preclinical validation	Qurbani et al. (2026), Zuberi et al. (2017)
Multiple adaptive pathways	Rational combination therapies	Simultaneously target complementary survival mechanisms	May reduce adaptive escape and improve antimicrobial activity	Potential antagonism, complex dosing and limited clinical evidence	Preclinical/experimental, interaction-dependent and largely non-clinical	Qurbani et al. (2026), Makabenta et al. (2023), Kortright et al. (2019)

Evidence-level terminology: Experimental- Predominantly *in vitro*/mechanistic evidence, Preclinical- Evidence from animal models, Early clinical- Human clinical evaluation, Clinical- Human efficacy/safety evidence.

## 6 Translational challenges and future perspectives

Although significant progress in knowledge has been made regarding the mechanisms of antimicrobial survival in biofilms, the transformation of such discoveries into clinically effective intervention is difficult. In clinical situations, biofilms are not as simple as the artificial models used in the laboratory, but instead form in the context of complex environments in which other microorganisms, the host immune response, tissue structures, exposure to antimicrobial agents, and anatomical location all play a role in their formation. Thus, caution needs to be used when extrapolating the findings of mechanisms identified in controlled experimental models to predict therapeutic outcomes in patients. Polymicrobial biofilms are a major challenge of translation. Numerous clinically relevant biofilms are polymicrobial, consisting of more than one species of bacteria, and sometimes fungi. In these communities, microorganisms communicate via cooperation, competition, extracellular environment modification, and signalling networks as well as metabolic exchange. One important form of interaction is metabolic cross-feeding, which plays a role in the survival of biofilms. One species may generate metabolic intermediates or degradation products that are useful to the growth or survival of another species and thus enable community members to inhabit ecological niches that would not be viable in the absence of the other species. At the community level, such interactions can alter the availability of nutrients, as well as metabolic activity and antimicrobial susceptibility. Microorganisms in polymicrobial biofilms do not only cooperate metabolically but can also share structures of the extracellular matrix. The products of one species’ matrix may act as structural support or change antimicrobial distribution for neighbouring species, enhancing community level resilience. However, the number of shared matrices varies depending on the species composition, environmental conditions and type of infection environment. Competition among microbes also affects polymicrobial biofilms. Microorganisms can compete for nutrients, create inhibitory compounds or disrupt signalling pathways, which affects the composition of the community and the antimicrobial response. Thus, the effect of microbial interactions can be either positive or negative depending on the ecological situation. Inter-species communication and horizontal gene transfer are various other factors affecting polymicrobial biofilm adaptation. There is the possibility that close proximity in biofilms could promote genetic exchange by transformation, conjugation or transduction and that this could contribute to the spread of resistance determinants. But the efficiency of gene transfer is influenced by a variety of factors such as donor/recipient compatibility, mobile genetic elements, environmental conditions and selective pressures. Thus, polymicrobial biofilms should be thought of as dynamic ecological systems, not as a collection of individual bacterial populations. Antimicrobial responses are a function of interactions between the microbial community, host status and environment. Thus, the results from monospecies models can give useful mechanistic information, but can be misleading in the prediction of the behaviour of complex clinical biofilms. The principal experimental models currently used to investigate biofilm-associated antimicrobial responses differ substantially in physiological complexity and translational relevance, as summarized in Table 6. Popular assays like static microtiter plate assays are useful for assessing biofilm biomass and antimicrobial effect but are unable to fully mimic the dynamic nature of host environments. Flow-cell and continuous-culture systems are more representative of nutrient flow and shear forces, but are still a simplification of tissue-associated infections. Biofilms associated with devices add other considerations such as materials, immune response, and chronic colonization. In addition, the traditional MIC assays with planktonic cultures are known to underestimate the tolerance of the antimicrobial activity of populations of biofilm-associated microorganisms. Hence, integration of complementary models representing structural, physiological, and ecological features of infection-associated biofilms is necessary for clinically meaningful evaluation. Future studies should thus include more complementary models, such as more advanced *in vitro* models, patient-derived biofilms, organoid models, and *in vivo* approaches, and not be based on a single platform. To gain insights into the contributions of individual bacterial states to survival and resistance emergence, single-cell approaches, spatial analysis, multi-omics technologies and evolutionary experiments are gradually emerging. These methods can help discover markers that can be used to predict treatment response and uncover vulnerabilities which are not visible from population-level analyses.

**Table 6:** Experimental models for studying biofilm-associated antimicrobial responses.

Model system	Major advantage	Major limitation	Translational relevance	References
Static microtiter biofilm assay	Simple, reproducible, high-throughput screening	Limited nutrient flow and environmental complexity	Mainly preliminary screening	Azeredo et al. (2017)
Flow-cell biofilm model	Incorporates fluid flow and dynamic growth conditions	Limited host interaction	Improved physiological relevance	Azeredo et al. (2017), Hall-Stoodley et al. (2004)
Continuous culture model	Enables long-term adaptation and evolution studies	Simplified compared with infections	Useful for evolutionary studies	Azeredo et al. (2017)
Polymicrobial biofilm models	Captures microbial interactions	More difficult to standardize	Higher relevance for chronic infections	Koo et al. (2017), Stacy et al. (2016)
Animal infection models	Includes host responses and pharmacology	Biological differences from humans	Important preclinical validation	Koo et al. (2017), Hall-Stoodley et al. (2004)
Patient-derived and advanced models	Better reflects clinical complexity	Technical and logistical challenges	Future translational direction	Azeredo et al. (2017), Moskowitz et al. (2004)

## 7 Conclusion

Biofilm-associated antimicrobial failure is a complex biological phenomenon where microbial physiology, ecology, regulation and evolution all play a role. While antimicrobial resistance is a large part of the reason for treatment failure, the reason for the survival of biofilm-associated populations cannot be solely attributed to genetically encoded antimicrobial resistance mechanisms. Rather, biofilms maintain their integrity through reversible physiological changes, phenotypic heterogeneity, persistence states, and in optimal selective conditions, emergence or acquisition of resistance determinants. Antimicrobial tolerance, persistence and resistance should be thought of as related but distinct biological phenomena. Tolerance and persistence allow bacterial populations to withstand antimicrobial exposure, and may not require heritable changes in susceptibility. Although these survival states may increase opportunities for resistance evolution by providing viable populations during selection, they are not an obligatory route to resistance. The shift from transient survival to stable resistance requires many factors such as the species of bacteria, the dynamics of antimicrobial exposure, the population structure and variation, and ecological interactions, as well as the availability of transferable resistance determinants. Biofilms are seen as a dynamic adaptive ecosystem not just as communities of resistant bacteria, which yields a more realistic picture of how to create effective interventions. Biofilm-mediated survival relies on structurally, physiologically and regulatory interconnected processes, which provide potential targets for therapy. The extracellular matrix organization, regulatory signalling, metabolic adaptation, persistence-associated pathways, efflux-associated responses and interactions with microbes may offer targets to improve antimicrobial activity and minimize the potential for evolutionary escape. But, most of the new anti-biofilm strategies are still experimental or pre-clinical, which means careful testing in clinically relevant models is needed. Future studies should go beyond simplifying interpretations of resistance and explore how ecological and physiological conditions impact evolutionary outcomes under antimicrobial treatment. Single-cell technologies, spatial approaches, multi-omics analyses, experimental evolution and clinically representative biofilm models will be key in identifying the predictive markers of treatment response, and designing evolution-informed therapies. Finally, the strategies that will be successful in the management of biofilm-associated infections will need to address not only the ability of bacteria to survive but also the adaptive processes that enable microbial communities to survive and evolve under antimicrobial selection.
